# Safety, pharmacokinetics, and pharmacodynamics of human interferon-α2b spray in healthy participants

**DOI:** 10.1128/aac.00686-25

**Published:** 2025-08-14

**Authors:** Wen-rui Zhang, Wei-zhe Jian, Yong-xing Chen, Yue-yuan Huang, Tian-yan Zhou, Xiao-qing Wen, Xi Luo

**Affiliations:** 1 Drug Clinical Trial Institution, The First Affiliated Hospital of Xiamen University 117892, Xiamen, Fujian, China; 2 School of Basic Medicine and Clinical Pharmacy, China Pharmaceutical University 56651, Nanjing, Jiangsu, China; 3 Department of Pharmaceutics, School of Pharmaceutical Science, Peking University 12465, Beijing, China; 4 School of Pharmaceutical Sciences, Xiamen University 12466, Xiamen, Fujian, China; Providence Portland Medical Center 25165, Portland, Oregon, USA

**Keywords:** human interferon-α2b spray, healthy participants, safety, pharmacodynamics, pharmacokinetics

## Abstract

The safety, pharmacokinetics (PK), and pharmacodynamics (PD) of multiple doses of human interferon (IFN)-α2b spray administered via nasal and oropharyngeal routes were evaluated in a randomized, double-blind, placebo-controlled study. Forty-eight healthy Chinese participants were randomized into three dose groups: low (450,000 IU/dose), medium (600,000 IU/dose), and high (750,000 IU/dose). In each group, 12 participants received the corresponding dose of IFN-α2b spray, and four participants received the placebo. All participants received a single nasal and oropharyngeal dose at 0 and 48 h, respectively, with the high-dose group receiving an additional topical abdominal skin dose at 96 h to assess skin irritation. Adverse events were observed and recorded throughout the trial. Plasma, nasal lavage fluid, and oral gargle samples were collected, with IFN concentration and interferon-inducible protein-10 level being determined as PK and PD indicators, respectively. The results suggest that IFN-α2b spray was safe and well-tolerated across all doses (450,000–750,000 IU) with no systemic exposure detected. PK/PD analysis showed a correlation between the PD index and the level of IFN exposure in nasal lavage fluid, while no significant correlation was observed in oral gargle samples. The IFN concentrations in all dose groups were higher than the half-maximal effective dose (ED_50_), suggesting that the high dose could be considered for subsequent clinical trials.

## INTRODUCTION

Respiratory infections remain among the top causes of global mortality, while viruses are the second most common cause of severe respiratory infections (1). Severe viral infections can cause acute respiratory failure and progress into acute respiratory distress syndrome (2, 3). The incidence of serious illness and mortality from respiratory infections is higher in high-risk groups such as the elderly, children, individuals with chronic underlying conditions, the obese, and pregnant women (4–7). Therefore, prompt treatment and prophylaxis in high-risk populations are necessary during viral respiratory infections or epidemics.

Interferon (IFN) is a cytokine with antiviral and immunomodulatory effects, and IFN-α2b is one of the isoforms of the α2 subclade of the α family of type I IFNs (8). The type I IFN response is an important part of antiviral innate immunity, in which host cells can release IFN after recognizing foreign nucleic acid signals from viruses, thereby interfering with viral replication in infected cells and also promoting the activation of specific immunity (9, 10). In addition, IFN also acts on neighboring uninfected cells, causing the expression of interferon-stimulated genes to further resist viral infection (11).

Gao et al. (12) demonstrated that nebulization with IFN-α2b is effective in preventing respiratory infections caused by the influenza virus, parainfluenza virus, and adenovirus. Spraying human IFN-α2b within 3 days of the first positive test for severe acute respiratory syndrome coronavirus 2 (SARS-CoV-2) shortened the viral shedding time of the SARS-CoV-2 Omicron variant (13). It has been shown that SARS-CoV-2 infects humans via the angiotensin-converting enzyme 2 (ACE2) receptor (14, 15). In the respiratory system, ACE2 receptor expression is highest in the nasal cavity and decreases down the lower respiratory tract. After SARS-CoV-2 invades the human body through the respiratory tract, the viral titer in nasal cavity epithelial cells is relatively high and gradually decreases in pharyngeal and bronchial cells, with the lowest titer in lung cells. These findings suggest that IFN-α2b may inhibit viral replication locally in the nasal cavity or oropharynx, thus effectively suppressing viral infection (16).

Currently, IFN is available in a variety of dosage forms, including injections, suppositories, gels, and topical sprays; however, no sprays have been approved for respiratory infections. In clinical practice, the nebulized inhalation of IFN is sometimes used to treat respiratory infections (17), allowing high local concentrations in the respiratory tract. However, the nebulization process requires special equipment and patient cooperation. The human IFN-α2b spray, developed from recombinant human IFN-α2b injection, delivers IFN as a solution-type spray to the nasal cavity and oropharynx, establishing a defense barrier at the infection site. This spray is easy to use and carry and may enhance the effectiveness of preventing and treating respiratory infections.

To date, no pharmacokinetic (PK) or pharmacodynamic (PD) studies of IFN-α2b spray have been reported. In this study, we performed the first PK/PD analysis and exposure-response (E-R) analysis of IFN-α2b spray, assessing the appropriateness of the current dosage regimen and efficacy indices. Our findings may facilitate the development of broad-spectrum antiviral drugs for respiratory infections and reduce the infection rate and severe disease risk.

## MATERIALS AND METHODS

### Participants

Eligible participants were healthy males or females aged 18–45 years (body mass index [BMI] 18.5–28 kg·m^−2^). Male participants were required to weigh ≥50 kg, and females ≥45 kg. Health status was defined as no evidence of active or chronic disease after a complete inquiry, physical examination, vital sign examination, and laboratory tests. Eligible female participants were required to have a negative pregnancy test during the screening period and up to 24 h before dosing. All participants were required to use strict non-pharmacological contraception from screening until 3 months after the end of the study and were prohibited from donating ova or sperm.

Exclusion criteria included (i) significant illness or infectious disease; (ii) history of substance abuse or drug use; (iii) allergy to the study drug; (iv) nasal/oropharyngeal mucosal lesions precluding safe sampling; (v) use of prescription medications, over-the-counter drugs, herbal products, or other specialized diets (e.g., grapefruit) that could affect the pharmacokinetics of the drug within 14 days before dosing; (vi) smoking (>5 cigarettes/day) or alcohol consumption (>14 units/week) within 3 months prior to screening; (vii) intake of chocolate, any caffeinated, alcoholic, or xanthine-rich foods or beverages in the 24 h before the administration of the trial drug; (viii) blood donation or blood loss exceeding 450 mL within 3 months prior to screening; (ix) participation in other clinical trials within 3 months prior to screening.

### Drugs and reagents

The IFN-α2b spray (500,000 IU/mL; Lot: SHI202212EP07) and placebo were provided by Xiamen Amoytop Biotech Co., Ltd. (China). Sample analyses were performed using microplate readers (Molecular Devices, USA). For IFN-α2b quantification, the following reagents were utilized: IFN-α2 HS Microplate, IFN-α2 HS Conjugate, and Streptavidin Polymer-HRP (R&D Systems, USA; Lot: P362628). For interferon-inducible protein 10 (IP-10) detection, the Human IP-10 Microplate and Human IP-10 Detection Antibody (Sino Biological, China; Lot: CW16MA0901) were employed.

### Study design and administration

This was a multi-dose, randomized, double-blind, placebo-controlled study in 48 healthy Chinese participants to evaluate the safety, PK, and PD of IFN-α2b spray administered via the nose and oropharynx.

After a 7-day screening period, participants who met the entry criteria entered the clinical site on day −1. The treatment period was designed with three dose levels: low (450,000 IU/dose), medium (600,000 IU/dose), and high (750,000 IU/dose). In view of nasal mucosal metabolism and the participants’ comfort, each dose level was divided into two sampling groups of eight participants, six of whom received the IFN-α2b spray, and two of whom received the placebo. All participants received the corresponding dose of IFN-α2b spray or placebo at 0 and 48 h, and the drug was administered simultaneously via the nose and oropharynx. In order to assess skin irritation, the high-dose group (750,000 IU/dose) was administered topically on the skin of the abdomen at 96 h (Table 1).

**TABLE 1:** Group assignment and drug administration^*a*^

Dosage (IU)	Sample size (*N* = 48)	Group of sampling	Sample size of IFN (*N* = 36)	Sample size of placebo (*N* = 12)	Time (h)		
					0	48	96
450,000	16	1	6	2	3 sprays in each nasal cavity + 3 sprays in oral pharynx	3 sprays in each nasal cavity + 3 sprays in oral pharynx	NA
		2	6	2			
600,000	16	1	6	2	4 sprays in each nasal cavity + 4 sprays in oral pharynx	4 sprays in each nasal cavity + 4 sprays in oral pharynx	NA
		2	6	2			
750,000	16	1	6	2	5 sprays in each nasal cavity + 5 sprays in oral pharynx	5 sprays in each nasal cavity + 5 sprays in oral pharynx	15 sprays to the abdominal region
		2	6	2			

^*a*^ *N*, number of participants; NA, not applicable.

Samples of plasma, nasal lavage fluid, and oral gargles were collected during the trial according to the protocol. Vital signs and adverse events (AEs) were observed and recorded throughout the trial. Participants in the low-dose and medium-dose groups were observed until 96 h, and those in the high-dose group were observed until 144 h before they left the clinical site.

### Assessments

The nasal and oral cavities were the primary sites for IFN’s anti-infective effects. In this study, the concentration of IFN in nasal lavage fluid and oral gargles was used as the PK indicator, and the concentration of IP-10 was used as the PD indicator (18).

Plasma samples were collected 1 h before and 0.5, 1, 2, 3, 4, 5, 6, 7, 8, 12, 24, and 48 h after drug administration. Nasal lavage samples were collected 1 h before and 0.5, 1, 2, 3, 4, 6, 8, 12, 16, 20, 28, and 36 h after drug administration. Oral gargle samples were collected 1 h before and 0.25, 0.5, 1, and 2 h after drug administration. IFN concentrations and IP-10 levels were measured in nasal lavage fluid and oral gargle samples. In the PK analysis, only the first nasal lavage or oral gargle samples collected after dosing can accurately reflect drug exposure. To adequately characterize the PK profile, two sampling periods and two sampling groups were designed based on the principles and methodologies of population pharmacokinetics (PopPK) (Table S1).

NCI CTCAE version 5.0 was used to determine the severity and grading of safety indicators. Safety indicators included AEs and serious adverse events (SAEs), physical examination, vital signs, laboratory tests, and local reactions.

### Bioanalytical assays

Concentrations of IFN-α2b and IP-10 in nasal fluid, oral fluid, and plasma samples were quantified by enzyme-linked immunosorbent assay by Suzhou Guochen Biotechnology Co.

Anti-human IFN-α2 antibody was immobilized on a microtiter plate and incubated with calibration standards, blank samples, quality control samples, and samples to be tested, followed by detection of IFN-α2 antibody labeled with biotin and streptavidin horseradish peroxidase against biotin. When the sample to be tested contained IFN-α2b, the complex would be formed, and HRP catalyzed the chromogenic reaction, producing a blue product that turned yellow after adding the stop solution. The color intensity was positively correlated with the concentration of IFN-α2b. The quantitative range was between 1.250 and 80.000 pg·mL^−1^. The precision and accuracy for plasma samples were 4.1%–4.8% and 0.6%–5.2%, respectively. The precision and accuracy range from 4.3% to 7.4% and 4.2% to 8.5%, respectively, for nasal and oral samples.

IP-10 levels were measured using the method analogous to that employed for IFN quantification, with anti-IP-10 antibody. The quantitative range was between 25.000 and 1,600.000 pg·mL^−1^. The precision and accuracy ranged from 6.5% to 9.2% and −0.5% to 3.6%, respectively.

### Statistical analysis

Statistical analysis was performed using SAS software. AEs were coded using MedDRA, and the number of cases, number of instances, and incidence of AEs were calculated separately for each group. AEs were categorized and counted at both system organ classification (SOC) and preferred terminology (PT) levels.

In this study, the nonlinear mixed effects model was used to conduct the population analysis, with the first-order conditional estimation with interaction for parameter estimation. Inter-individual variability of parameters was described using an exponential error model (equation 1).


(1) $${Para}_{i}={Para}_{TV}\cdot \exp(\eta ),$$

where Para_*i*_ and Para_TV_ are individual and population typical values of a parameter, *η* conforms to a normal distribution with mean 0 and standard deviation *ω*.

The summation (equation 2), proportional (equation 3), and mixed (equation 4) residual error models were tried to correct the fitting results, respectively.


(2) $$Obs=Pred+\varepsilon_{1},$$

(3) $$Obs=Pred\cdot (1+\varepsilon_{2}),$$

(4) $$Obs=Pred\cdot (1+\varepsilon_{2})+\varepsilon_{1},$$

where Obs and Pred are the measured value and model-predicted value of drug concentration or PD index, and *ε*_1_ and *ε*_2_ represent additive and proportional errors, respectively. All of them conform to the normal distribution with mean 0 and standard deviation *σ*.

Model accuracy was evaluated using objective function values, Akaike’s information criterion, reasonableness and relative standard error (RSE) of parameters, goodness of fit (GOF), and individual fit plots. Bootstrap resampling (1,000 times) was used to assess model robustness. Visual predictive checks (VPCs) with 1,000 simulations were used to evaluate model predictive performance.

## RESULTS

### Baseline and participant characteristics

A total of 48 healthy participants were enrolled at the First Affiliated Hospital of Xiamen University. All 48 participants completed the study and were included in the PK, PD, and safety analyses. Baseline characteristics were similar between the treatment and placebo groups (Table 2).

**TABLE 2:** Baseline demographics of participants^*a*^^,^^*b*^

Characteristics	450,000 IU		600,000 IU		750,000 IU		Pooled IFN (*N* = 36)	Pooled placebo (*N* = 12)	Overall (*N* = 48)
	IFN (*N* = 12)	Placebo (*N* = 4)	IFN (*N* = 12)	Placebo (*N* = 4)	IFN (*N* = 12)	Placebo (*N* = 4)			
Age (years), median (range)	26.5 (19, 35)	26.0 (22, 30)	30.5 (21, 38)	24.5 (18, 33)	29.5 (23, 37)	27.5 (21, 36)	29.0 (19, 38)	25.0 (18, 36)	27.5 (18, 38)
Male, *n* (%)	10 (83.3)	3 (75.0)	11 (91.7)	3 (75.0)	7 (58.3)	3 (75.0)	28 (77.8)	9 (75.0)	37 (77.1)
Height (cm), mean (SD)	165.00 (5.218)	167.00 (9.772)	168.63 (8.539)	165.38 (8.518)	160.92 (8.699)	164.00 (7.832)	164.85 (8.090)	165.46 (8.013)	165.00 (7.990)
Weight (kg), mean (SD)	62.33 (9.044)	68.95 (8.453)	65.65 (11.766)	58.18 (5.986)	61.19 (9.527)	61.65 (11.201)	63.06 (10.071)	62.93 (9.245)	63.02 (9.774)
BMI (kg·m^−2^), mean (SD)	22.853 (2.7960)	24.708 (1.9063)	22.948 (2.7395)	21.280 (1.6135)	23.569 (2.5069)	22.780 (2.3792)	23.123 (2.6257)	22.923 (2.3220)	23.073 (2.5306)

^*a*^ *N*, number of participants.

^*b*^ Data are presented as median (range) for age; *n* (%) for genders; and mean ± SD for all other parameters.

### Safety

All 48 participants were included in the safety assessments for nasal and oropharyngeal administration, and 16 participants in the high-dose group were included in the safety assessments for topical abdominal administration. There were no SAEs and no study discontinuations, withdrawals, or dose adjustments due to AEs.

#### Transnasal and oropharyngeal administration

The incidence of AEs was 38.9% (14/36) in the treatment group and 33.3% (4/12) in the placebo group. The incidence was similar across dose groups, with one drug-related AE (urinary tract infection) reported in the high-dose group. The incidence of drug-related AEs, as determined by the investigators, was 25.0% (9/36) in the treatment group and 25.0% (3/12) in the placebo group. All drug-related AEs were grade 1 and resolved spontaneously without treatment. The incidence of drug-related AEs in the low-dose group was 25.0% (3/12), with one case of rash and two cases of elevated blood bilirubin. The incidence of drug-related AEs in the middle-dose group was 33.3% (4/12), including one case of elevated blood bilirubin, one case of elevated blood creatinine, and two cases of elevated blood cholesterol. The incidence of drug-related AEs in the high-dose group was 25.0% (3/12), including two cases of alanine aminotransferase elevation and one case of heart rate elevation (Table 3).

**TABLE 3:** Summary of TEAEs following nasal and oral administration^*b*^

SOC/PT	Treatment groups (IFN)								Placebo (*N* = 12)	
	450,000 IU (*N* = 12)		600,000 IU (*N* = 12)		750,000 IU (*N* = 12)		Total (*N* = 36)			
	Cases (%)	Events	Cases (%)	Events	Cases (%)	Events	Cases (%)	Events	Cases (%)	Events
**Any TEAE^*a*^**	**6** (**50.0**)^*c*^	**8**	**5** (**41.7**)	**5**	**3** (**25.0**)	**4**	**14** (**38.9**)	**17**	**4** (**33.3**)	**9**
**Investigations**	**5** (**41.7**)	**6**	**5** (**41.7**)	**5**	**2** (**16.7**)	**3**	**12** (**33.3**)	**14**	**4** (**33.3**)	**7**
Increased blood bilirubin	2 (16.7)	2	1 (8.3)	1	0	0	3 (8.3)	3	2 (16.7)	2
Increased alanine aminotransferase	0	0	0	0	2 (16.7)	2	2 (5.6)	2	0	0
Urinary casts detected	2 (16.7)	2	0	0	0	0	2 (5.6)	2	0	0
Increased blood cholesterol	0	0	2 (16.7)	2	0	0	2 (5.6)	2	1 (8.3)	1
Positive urine leukocytes	0	0	1 (8.3)	1	0	0	1 (2.8)	1	2 (16.7)	2
Abnormal urine cytology	1 (8.3)	1	0	0	0	0	1 (2.8)	1	0	0
Positive bacterial test	1 (8.3)	1	0	0	0	0	1 (2.8)	1	2 (16.7)	2
Increased heart rate	0	0	0	0	1 (8.3)	1	1 (2.8)	1	0	0
Increased blood creatinine	0	0	1 (8.3)	1	0	0	1 (2.8)	1	0	0
**Infections**	**0**	**0**	**0**	**0**	**1** (**8.3**)	**1**	**1** (**2.8**)	**1**	**0**	**0**
Urinary tract infection	0	0	0	0	1 (8.3)	1	1 (2.8)	1	0	0
**Psychiatric disorders**	**1** (**8.3**)	**1**	**0**	**0**	**0**	**0**	**1** (**2.8**)	**1**	**0**	**0**
Fear of injection	1 (8.3)	1	0	0	0	0	1 (2.8)	1	0	0
**Skin disorders**	**1** (**8.3**)	**1**	**0**	**0**	**0**	**0**	**1** (**2.8**)	**1**	**0**	**0**
Rash	1 (8.3)	1	0	0	0	0	1 (2.8)	1	0	0
**Vascular disorders**	**0**	**0**	**0**	**0**	**0**	**0**	**0**	**0**	**1** (**8.3**)	**2**
Hypotension	0	0	0	0	0	0	0	0	1 (8.3)	2

^*a*^ TEAE, treatment-emergent adverse event.

^*b*^ Data are presented as number of cases (percentage)/number of events. Percentages were calculated based on the total subjects in each group.

^*c*^ Bold values indicate summary data for the corresponding System Organ Class.

#### Localized transabdominal dermal administration

The incidence of AEs was 25.0% (3/12) in the treatment group and 75.0% (3/4) in the placebo group, all of which were grade 1 and resolved spontaneously without treatment. No drug-related AEs occurred in the treatment group (Table 4).

**TABLE 4:** Summary of TEAEs following topical abdominal administration^*b*^

SOC/PT	IFN (750,000 IU) (*N* = 12)		Placebo (750,000 IU) (*N* = 4)		Total (*N* = 16)	
	Cases (%)	Events	Cases (%)	Events	Cases (%)	Events
**Any TEAE^*a*^**	**3** (**25.0**)^*c*^	**6**	**3** (**75.0**)	**5**	**6** (**37.5**)	**11**
**Investigations**	**2** (**16.7**)	**5**	**3** (**75.0**)	**4**	**5** (**31.3**)	**9**
Positive urine leukocytes	2 (16.7)	2	0	0	2 (12.5)	2
Urinary casts detected	1 (8.3)	1	2 (50.0)	2	3 (18.8)	3
Abnormal urine cytology	1 (8.3)	1	0	0	1 (6.3)	1
Positive bacterial test	1 (8.3)	1	0	0	1 (6.3)	1
Urinary ketones detected	0	0	1 (25.0)	1	1 (6.3)	1
Elevated blood cholesterol	0	0	1 (25.0)	1	1 (6.3)	1
**Skin disorders**	**1** (**8.3**)	**1**	**1** (**25.0**)	**1**	**2** (**12.5**)	**2**
Erythema	1 (8.3)	1	1 (25.0)	1	2 (12.5)	2

^*a*^ TEAE, treatment-emergent adverse event.

^*b*^ Data are presented as number of cases (percentage)/number of events. Percentages were calculated based on the total subjects in each group.

^*c*^ Bold values indicate summary data for the corresponding System Organ Class.

### Pharmacokinetics

This study utilized IFN concentrations in samples to characterize the pharmacokinetic profile of the IFN spray. In blood samples, 97% of the IFN concentration data were below the lower limit of quantification, indicating that topical administration of human IFN-α2b spray did not result in systemic IFN exposure. This finding aligns with the low incidence of systemic adverse reactions observed in practice.

To assess the effect of multiple lavages on IFN concentrations, we compared the trend of data from “first lavage” and “non-first lavage” (Fig. S1a). Results revealed that repeated lavages significantly affected IFN concentrations in nasal lavage fluid, with lower observed values in non-first lavage samples. Therefore, only the “first lavage” observations were used to analyze IFN concentrations in the samples.

**Fig 1:**
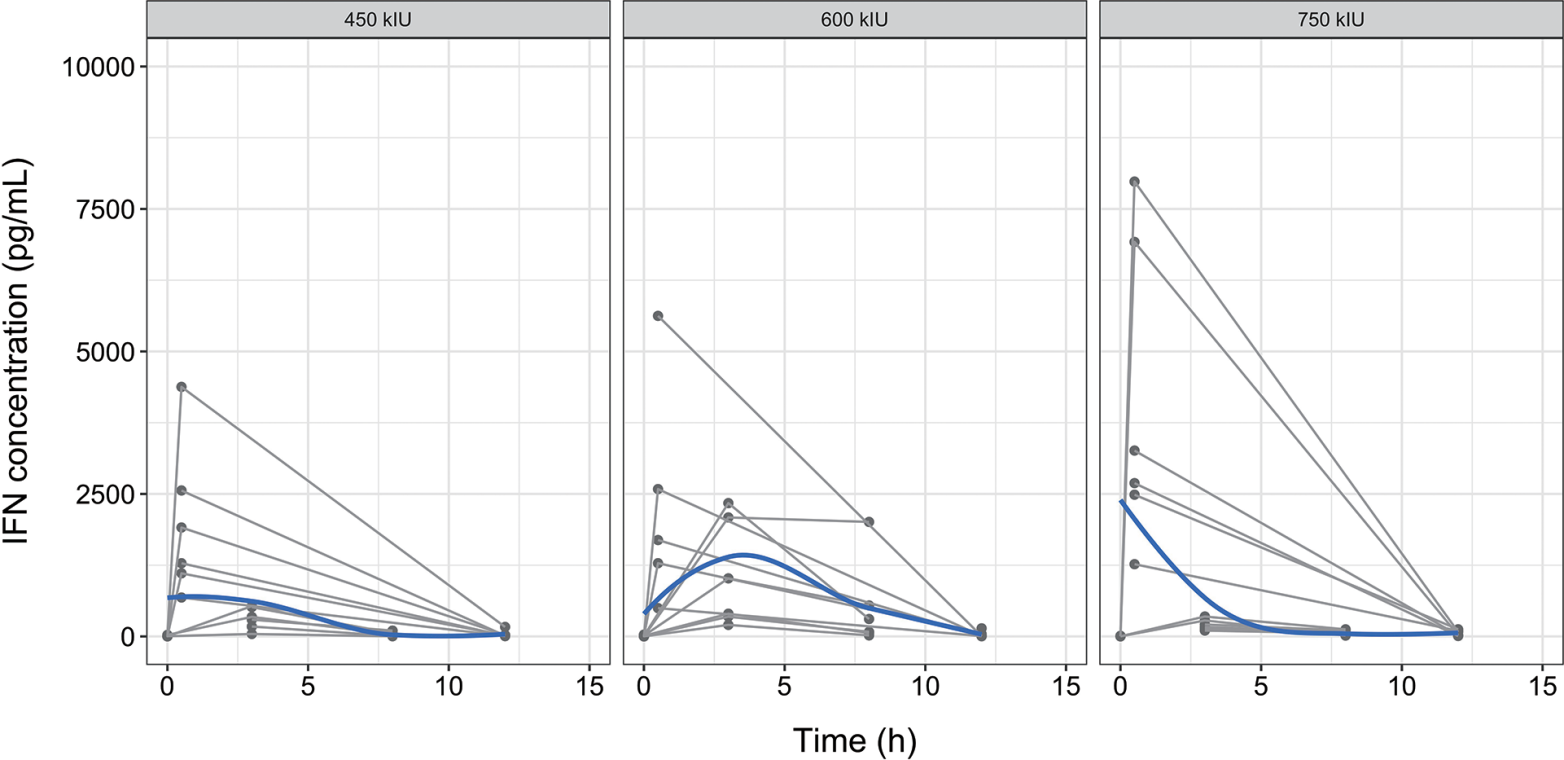
Concentration-time curve of IFN in nasal lavage fluid. The blue solid lines represent population trend lines. The gray solid lines indicate individual participant profiles.

As shown in the nasal lavage IFN concentration-time plot (Fig. 1), exposure to nasal lavage IFN increased with the administered dose. In the logarithmized longitudinal IFN concentration-time plot for nasal lavage fluid (Fig. S2), the IFN concentration decreased exponentially over time, consistent with a first-order elimination kinetic process.

**Fig 2:**
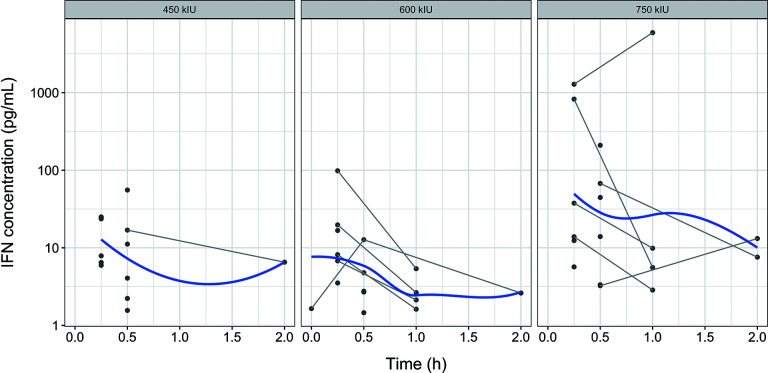
Concentration-time curve of IFN in oral gargles. The blue solid lines represent population trend lines. The gray solid lines indicate individual participant profiles.

As shown in the IFN concentration-time profile of oral gargle fluid (Fig. 2), IFN concentrations were relatively similar across the three dose groups, with a few higher concentration observations in the high-dose group. The IFN concentrations in oral gargle fluid also followed first-order elimination kinetics.

### Pharmacodynamics

Blood levels of β2-microglobulin, an indicator of PD for some diseases treated with IFN, were examined after topical administration in the high-dose group. No significant changes were observed following topical administration (Fig. S3). These findings suggested that topical administration of IFN-α2b spray did not result in systemic exposure, aligning with the safety assessments and pharmacokinetic results.

In our study, IP-10 concentrations were used to evaluate pharmacodynamic effects. Similarly, to assess the impact of repeated lavages on IP-10 levels, we compared trends between “first lavage” and “non-first lavage” data (Fig. S1b). Results showed that repeated lavages had minimal influence on IP-10 concentrations. Therefore, IP-10 observations from non-first lavage were retained in subsequent analyses.

**Fig 3:**
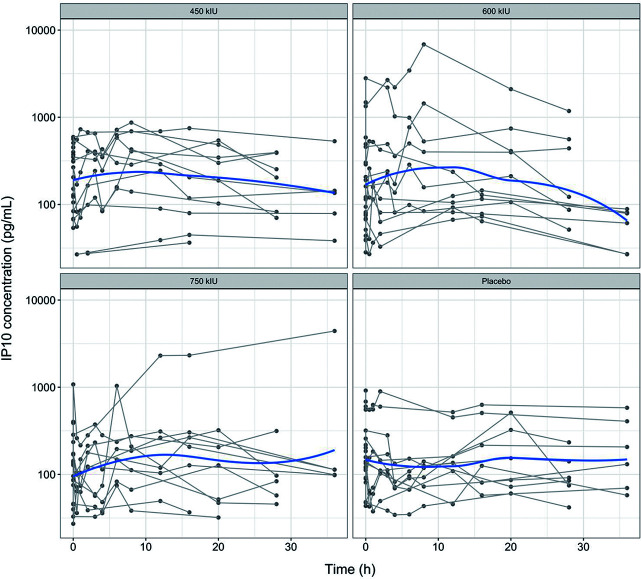
Concentration-time curve of IP10 in nasal lavage fluid. The blue solid lines represent population trend lines. The gray solid lines indicate individual participant profiles.

**Fig 4:**
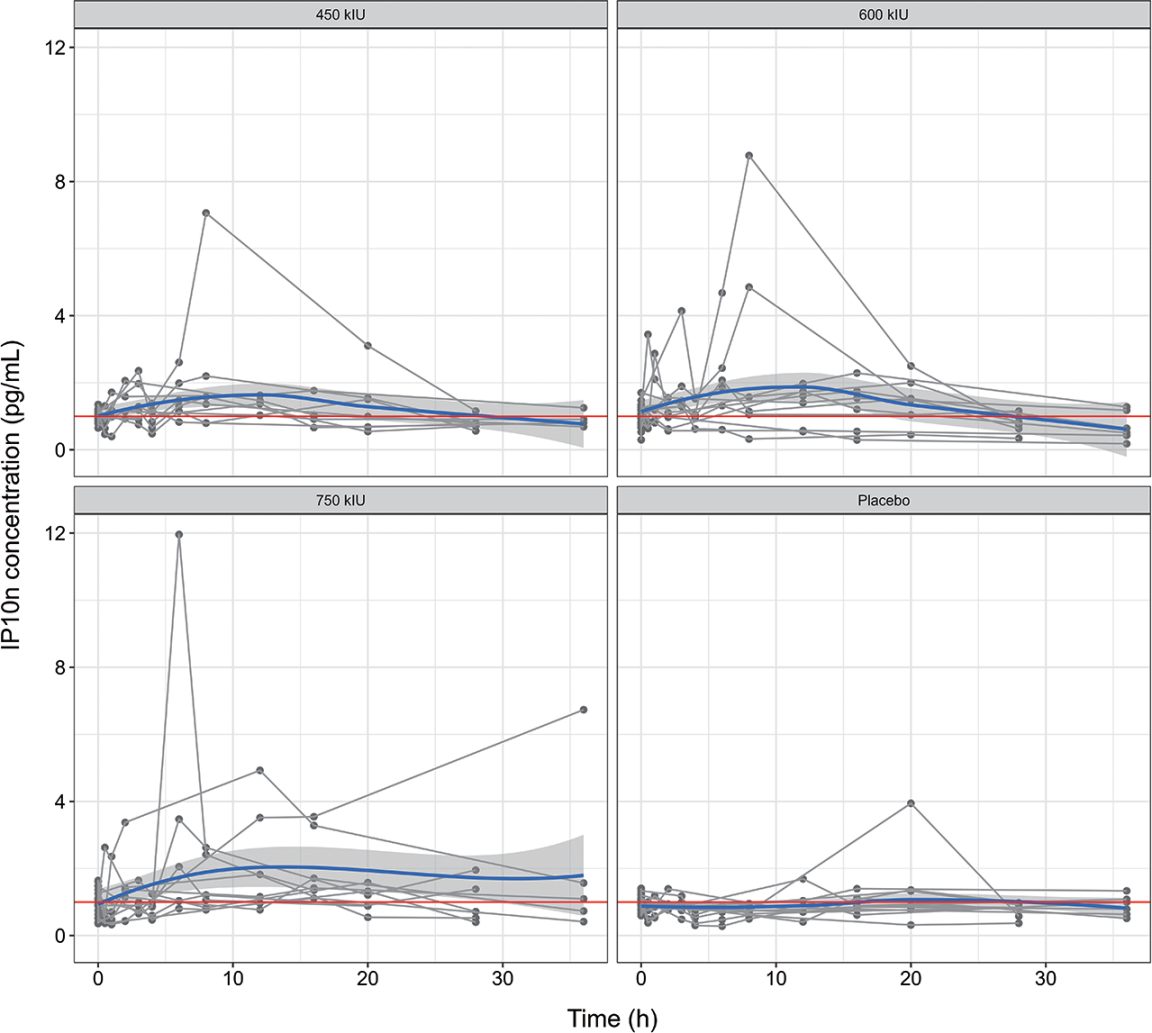
Concentration-time curve of IP10_*n*_ in nasal lavage fluid. The blue bold solid line represents the population trend, while the gray thin solid lines depict individual trajectories.

Nasal lavage IP-10 concentration-time plots showed (Fig. 3) that IP-10 concentrations increased after administration, reached a maximum around 10 h, and then returned to baseline values. There were no significant changes in IP-10 levels in the placebo group. Inter-individual variability in nasal IP-10 concentrations was high, with the distribution of concentrations in the three dose groups highly overlapping. This was similar to the pattern of the normalized nasal IP10_*n*_ concentration-time plots (Fig. 4). (IP10_*n*_ is corrected for IP10 based on the individual participant’s baseline value using the correction formula: IP10_*n*_= IP10/IP10_Base_).

As shown in the IP-10 concentration-time profile of oral gargle fluid (Fig. S4), IP-10 concentrations in the oral cavity increased after administration and remained elevated until the end of sampling. However, IP-10 levels in the control group also demonstrated an upward trend.

### PK/PD and E-R analysis

#### PK/PD analysis

**Fig 5:**
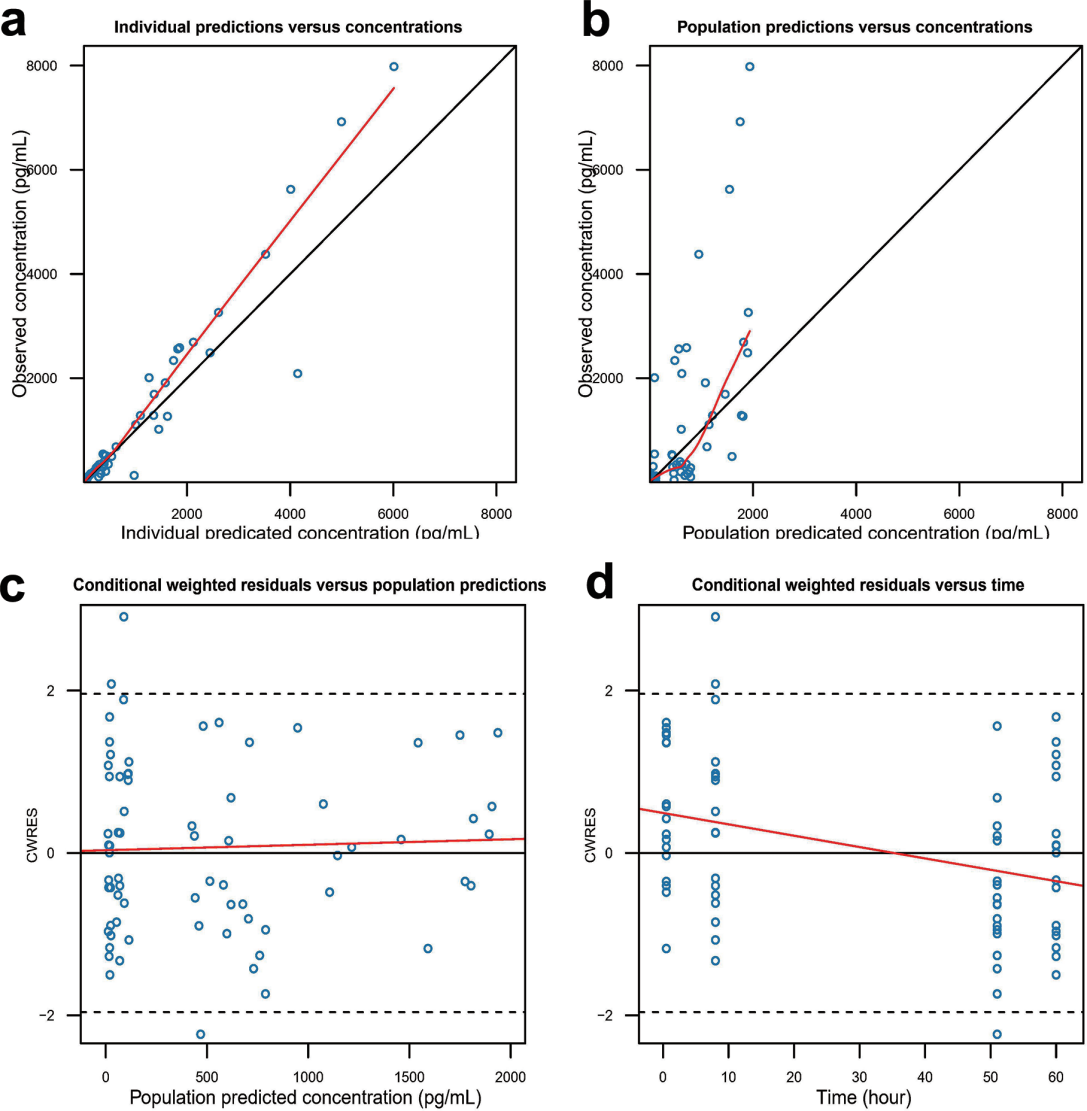
GOF plots for the PopPK model of IFN in nasal lavage fluid. (**a**) Observed vs individual predicted concentrations; (**b**) observed vs population predicted concentrations; (**c**) conditional weighted residuals (CWRES) vs population predicted concentrations; (**d**) CWRES vs time.

**Fig 6:**
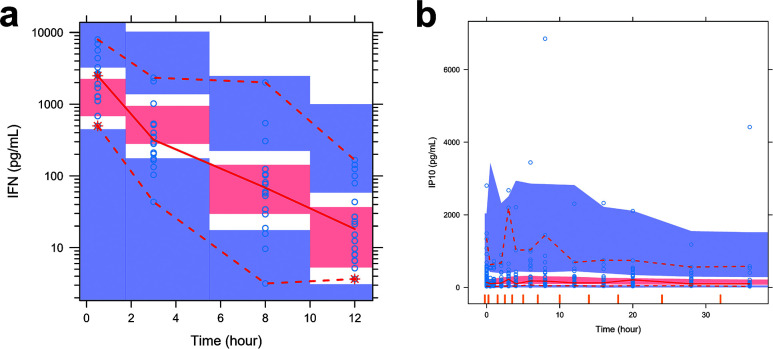
VPC plot for the PopPK and PK/PD model of IFN in nasal lavage fluid. (**a**) PopPK model; (**b**) PK/PD model. The blue circles refer to observations. The red solid line is the median line of observations, and the red shaded area stands for its 90% prediction interval. The red dashed lines are the 2.5% and 97.5% percentiles of the observations, and the blue shaded areas stand for their 90% prediction intervals, respectively.

A one-compartment model with instantaneous administration and first-order elimination was used to describe the dynamic changes of IFN concentration in nasal lavage (Fig. S5a). The model parameters and bootstrap results are shown in Table S2. The model parameters were reasonable, and the RSE of the parameters met the requirements for estimation precision. The typical values of the estimated parameters and the median values of the parameters obtained by the bootstrap method were similar, and all of them fell within the 95% confidence intervals, indicating good model stability. In addition, the nasal lavage PopPK model met the criteria in both the GOF plot (Fig. 5) and the VPC (Fig. 6a), confirming the model’s precision and predictive ability. Based on the typical values of the nasal PopPK model parameters and inter-individual variability, 1,000 virtual participants were generated to simulate the dynamic changes of IFN concentration in nasal lavage fluid receiving 450,000, 600,000, and 750,000 IU/dose, respectively (Fig. S6a).

**Fig 7:**
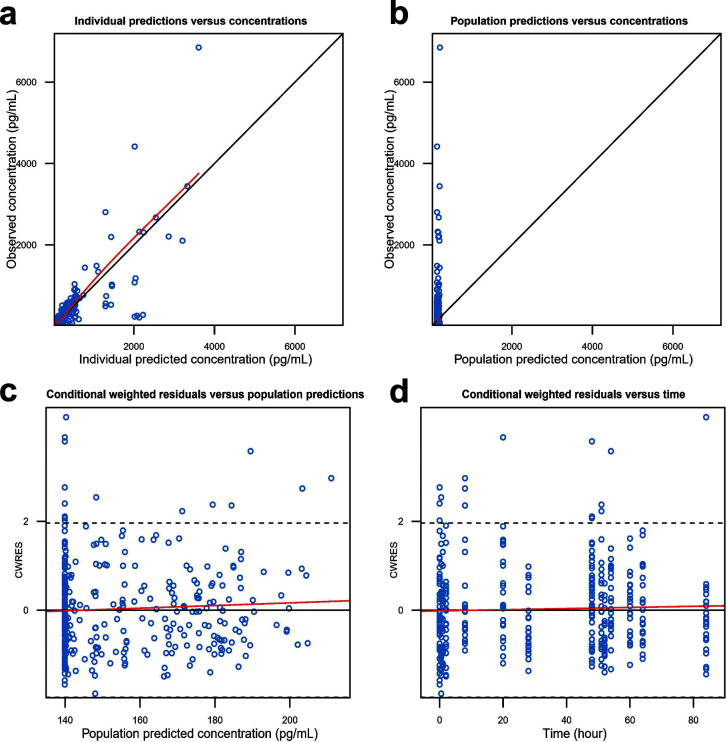
GOF plots for the PK/PD model of IFN in nasal lavage fluid. (**a**) Observed vs individual predicted concentration; (**b**) observed vs population predicted concentrations; (**c**) CWRES vs population predicted concentration; (**d**) CWRES vs time.

After establishing the PopPK model of nasal lavage fluid, the dynamic changes of IFN concentration were simulated for each participant based on the PopPK individual parameter values estimated by the model. A PK/PD model was further developed using the indirect response model to describe the increase in IP-10 levels after the administration (Fig. S5b). The model parameters and bootstrap results are shown in Table S3. The typical values of the parameter estimation were similar to the median values of the parameters obtained by the bootstrap method, and all of them fell within the 95% confidence interval, indicating good model stability. The nasal lavage PK/PD model also met the criteria in both GOF (Fig. 7) and VPC (Fig. 6b), confirming its accuracy and predictive ability. The parametric results of the nasal PK/PD model showed that the median IP-10 value before administration was 140 pg·mL^−1^, and the IP-10 level increased up to 68.2% after IFN administration. Exposure to IFN from nasal lavage increased with dose, with a large interindividual variability and overlap in the exposure ranges between doses. IFN concentrations were much higher than EC_50_ in all dose groups, and the upregulation of IP-10 was nearly saturated at all three doses. Based on the typical values of the parameters of the nasal PK/PD model and the interindividual variability, 1,000 virtual participants were generated to simulate the dynamics of IP-10 concentration in the nasal lavage fluid receiving different doses of the test drug (Fig. S6b). The extent of IP-10 increase in the three dose groups was similar but differed from the control group.

#### E-R analysis

The relationship between IFN exposure and IP-10 levels in nasal lavage following the use of IFN-α2b spray was analyzed. In this study, the relationship between PK and PD indicators was analyzed using the area under the concentration-time curve (AUC) from time 0 to ∞ and the area under the effect-time curve (AUEC) from time 0 to the last time point sampled. Given that the IFN concentration decreased exponentially over time, a linear mixed effects model was used to fit the logarithmized IFN concentrations with equation 5. Individual parameters were obtained by model fitting, and the individual AUC of IFN was calculated based on the values of individual parameters, as shown in equation 6. The trapezoidal method was used to calculate the area under the effect-time curve for IP-10 and IP-10_*n*_ as AUEC and AUEC_*n*_, respectively. In view of the total sampling time varying among participants, the individual AUEC and AUEC_*n*_ were corrected using the individual length of total sampling time (*τ*) using equations 7 and 8. After obtaining the individual AUC and AUEC/*τ* and AUEC_n_/*τ* for nasal lavage, the E-R relationship for IFN-α2b spray was analyzed by linear modeling with equations 9 and 10.


(5) ln⁡IFN=Intercept−Slope×Time,

(6) $$AUC=\int_{0}^{+\infty }e^{Intercept}\cdot e^{-Slope\cdot t}dt=\frac{e^{Intercept}}{Slope},$$

(7) $$\frac{AUEC}{\tau }=\frac{1}{\tau }\int_{0}^{\tau }IP10\left(t\right)dt,$$

(8) $$\frac{{AUEC}_{n}}{\tau }=\frac{1}{\tau }\int_{0}^{\tau }IP10n\left(t\right)dt,$$

(9) $$\frac{AUEC}{\tau }=\beta_{0}+\beta_{1}\times AUC,$$

(10) $$\frac{{AUEC}_{n}}{\tau }=\beta_{0}+\beta_{1}\times AUC,$$

where *β*_0_ is the intercept term, and *β*_1_ is the regression coefficient of the IFN concentration-time curve AUC.

**Fig 8:**
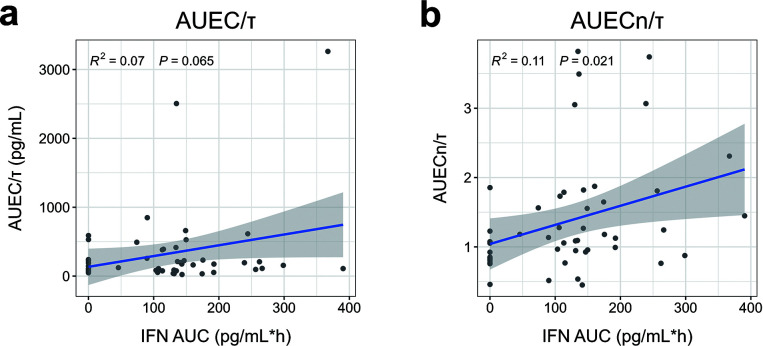
ER analysis of efficacy indicators in nasal lavage fluid to IFN exposure. (**a**) AUEC/τ vs IFN AUC (*R*^2^ = 0.07, *P* = 0.065); (**b**) AUECn/τ vs IFN AUC (*R*^2^ = 0.11, *P* = 0.021). IFN AUC, the area under the IFN concentration-time curve from time 0 to ∞ ; AUEC, the area under the IP10 concentration-time curve from time 0 to ∞; AUEC_*n*_, the area under the IP10*_n_* concentration-time curve from time 0 to ∞; and *τ*, the total sampling duration.

**Fig 9:**
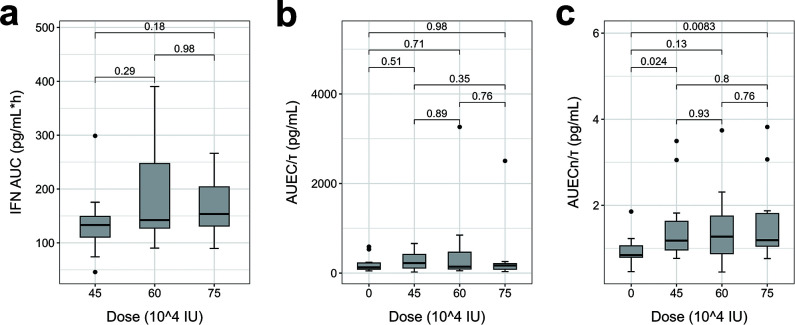
AUC of IFN concentration-time curve and efficacy indicators (AUEC/*τ*, AUEC_*n*_/*τ*) in nasal lavage fluid across different dose groups. (**a**) IFN AUC; (**b**) AUEC/τ; and (**c**) AUEC_*n*_/*τ*. Box lines (top to bottom): Q3, median, and Q1. Outliers: >Q3 + 1.5 IQR or <Q1 − 1.5 IQR (solid points). Whiskers: non-outlier range [min, max].

The parameter estimates of the E-R relationship model for the efficacy indicators (AUEC/*τ*, AUEC_*n*_/*τ*) are shown in Table S4. E-R analysis showed that AUEC/*τ* tended to increase with increasing IFN exposure (*P* = 0.065). Furthermore, an E-R relationship was observed between AUEC_*n*_/*τ* and IFN exposure (*P* = 0.021) (Fig. 8). Nevertheless, IFN exposure showed comparable levels across dose groups (*P* > 0.05). Of particular note, the IP10_*n*_-associated AUEC_*n*_/*τ* differed between the partial administration group and placebo group (*P* < 0.05) (Fig. 9).

Using the same modeling and ER analysis methods, we evaluated the relationship between IFN exposure and IP-10 levels in oral gargle fluid. Results demonstrated that oral gargle AUEC/*τ* and AUEC_*n*_/*τ* exhibited an upward trend with increasing IFN exposure, though this association did not reach statistical significance (*P* > 0.05). Comparative analysis revealed no detectable inter-group differences in IFN AUC, AUEC/*τ*, or AUEC_*n*_/*τ* across dose groups (*P* > 0.05).

## DISCUSSION

A randomized, double-blind, placebo-controlled trial was conducted to evaluate the safety, PK, and PD profiles of IFN-α2b spray administered across different treatment groups. Based on the levels of IFN or IP-10 in clinical samples, such as nasal lavage, oral gargle, and plasma collected during the study, PK/PD analyses and E-R analyses were performed, and AEs were observed and recorded throughout the trial. Regarding the design of plasma sampling points, taking into account the limited systemic absorption of the drug into the body circulation, the 12 blood sampling points after drug administration were evenly distributed across different sampling groups in order to protect the safety of the participants as much as possible.

Candidate PD indicators for early clinical trials are interferon-related signaling pathway proteins, including protein kinase R, oligoadenylate synthetase, neopterin, IP-10, etc. (19–21). Finally, IP-10 was selected as a PD indicator for early clinical trials. IP-10 is a chemokine with a molecular weight of 10 kDa induced by IFN and exerts chemotaxis by interacting with the receptor CXCR3 (22, 23). It has been reported that an early increase in IP-10 level may aid in the elimination of SARS-CoV-2 (24), making IP-10 a feasible PD indicator to assess whether the dose of IFN-α2b spray is sufficient to activate the IFN pathway. However, the dose range studied (450,000–750,000 IU/dose) may have approached the plateau phase of the dose-response curve, resulting in limited differences in PK and PD parameters between doses. As IP-10 is a downstream biomarker and the study population consisted of healthy participants, its trend across doses may not be linear. Nevertheless, this study demonstrated preliminary differences in IP-10 levels compared to placebo, supporting its potential as a future efficacy indicator. Subsequent studies will enroll patients and focus more on evaluating clinical efficacy endpoints (e.g., viral load, symptom scores, and time to recovery) alongside potentially more sensitive local biomarkers to better determine the optimal dose.

The PK/PD analysis explored the real-time mathematical relationship between IFN concentrations and IP-10 levels, while the E-R analysis aimed to find the overall relationship between IFN and IP-10 exposure without model assumptions, which was much more objective. The results of the PK/PD analysis showed that the IFN concentrations in nasal lavage were all much higher than EC_50_, and the degree of increase in IP-10 was approximately similar between different dose groups but differed from the placebo group. In the E-R analysis, AUEC_*n*_/*τ* was significantly higher in partially dosed groups compared to the placebo group, and a significant E-R relationship was observed between AUEC_*n*_/*τ* and IFN exposure. This suggested that the higher IFN exposure in nasal lavage within a given range led to a greater increase in IP-10 from baseline. However, no significant differences in IFN exposure were observed between different doses, possibly due to saturation of absorption in the limited nasal mucosa area or assay method variability masking dose-related differences. The high inter-individual variability in PK and PD data may require future analysis with larger sample sizes.

IFN exposure and IP-10 levels in oral gargles at different doses were more similar, with a shorter half-life of IFN than in the nasal cavity and greater inter-individual variability. This may be due to the fact that the oral environment is influenced by eating, drinking, and salivary swallowing, leading to a greater inter-individual variability in the oral cavity than in the nasal cavity. Concentrations of IP-10 in oral gargles were close to the lower limit of quantification, making accurate quantification of IFN and IP-10 in oral gargle challenging. E-R analysis showed that the IP-10 level-time curves of the placebo group and the drug administration group in oral gargles were relatively consistent, and the AUEC/*τ* and AUEC_*n*_/*τ* were relatively similar, and no obvious drug effect was observed, which might be related to the sensitivity of the IP-10 analysis and the detection error. In the PK/PD modeling study, the model assumed that IP-10 in the placebo group remained at baseline levels, which was different from the actual observation, potentially limiting the model’s ability to predict IP-10 dynamics in the placebo group. Therefore, no significance was found in the oral gargle PK/PD correlation and E-R analysis.

Within the dose range of this study (450,000–750,000 IU/dose), drug-related adverse events occurred similarly to previous studies (13, 25), and the overall safety risk of the drug was low. The results of topical administration in the high-dose group also suggested that the drug was not significantly irritating. Among all dose groups, the 750,000 IU/dose group demonstrated a favorable safety profile. The incidence and severity of AEs in this group were not significantly higher than those in other groups. Although the IP-10 response showed no clear dose gradient, the 750,000 IU/dose achieved relatively high IFN levels in nasal lavage fluid. As a locally administered spray, we aim to maximize respiratory tract coverage. Therefore, based on the available PK and safety data, we selected the 750,000 IU/dose for the next phase of the study to potentially demonstrate efficacy in a patient population. Exploring doses slightly below 750,000 IU/dose remains a future direction for dose optimization.

### Conclusions

In this study, the safety of IFN-α2b spray was evaluated, and PK/PD correlation and E-R analysis were performed. The results showed that there was a correlation between the efficacy indexes and the IFN exposure level in the nasal lavage, while no significant correlation was observed in the oral gargles. IP-10 may be an important indicator for evaluating the efficacy of IFN-α2b sprays. IFN-α2b spray was safe and well tolerated when administered as a single dose of 450,000–750,000 IU/dose nasally or oropharyngeally, and the dose of 750,000 IU/dose may be selected for subsequent clinical trials.

## SUPPLEMENTAL MATERIAL

Supplemental material aac.00686-25-s0001.docx Tables S1 to S4; Fig. S1 to S6.

ASM does not own the copyrights to Supplemental Material that may be linked to, or accessed through, an article. The authors have granted ASM a non-exclusive, world-wide license to publish the Supplemental Material files. Please contact the corresponding author directly for reuse.
