# Supplementary material for: Safety, pharmacokinetics, and pharmacodynamics of human interferon-α2b spray in healthy participants
Source: Antimicrob Agents Chemother. 2025 Aug 14;69(10):e00686-25. doi: 10.1128/aac.00686-25 (PMC12486823; doi:10.1128/aac.00686-25)

**Safety, pharmacokinetics, and pharmacodynamics of human interferon-α2b spray in healthy participants**

**Running Head：Safety, PK, and PD of IFN-α2b spray**

**All authors:**

Wen-rui Zhang^1,2*^, Wei-zhe Jian^3*^, Yong-xing Chen^1,4^, Yue-yuan Huang^1^, Tian-yan Zhou^3^, Xi Luo^1^✉, Xiao-qing Wen^1^✉

^1^ Drug Clinical Trial Institution, The First Affiliated Hospital of Xiamen University, Xiamen 361003, Fujian, China

^2^ School of Basic Medicine and Clinical Pharmacy, China Pharmaceutical University, Nanjing 211198, Jiangsu, China

3 Department of Pharmaceutics, School of Pharmaceutical Science, Peking University, Beijing 100871, China

4 School of Pharmaceutical Sciences, Xiamen University, Xiamen 361002, Fujian, China

***These authors contributed equally：**Wen-rui Zhang, Wei-zhe Jian

**✉Correspondence:** Xi Luo ([luoxi2999@163.com](mailto:luoxi2999@163.com)) or Xiao-qing Wen (wxqgcp@sina.com)

**Number of Supplementary Tables:** 4

**Number of Supplementary Figures:** 6

**Supplementary Table S1** Sampling schedule

| Study Period | Time | Plasma PK | | Nasal PK | | Nasal PD | | Oral PK | | Oral PD | |
| --- | --- | --- | --- | --- | --- | --- | --- | --- | --- | --- | --- |
|  |  | 1 | 2 | 1 | 2 | 1 | 2 | 1 | 2 | 1 | 2 |
| Ⅰ | -1 h ~ 0 h | × | × | × | × | × | × | × | × | × | × |
|  | 0 h | Dose Administration | | | | | | | | | |
|  | 0.25 h |  |  |  |  |  |  | × |  | × |  |
|  | 0.5 h | × |  | × |  | × |  |  | × |  | × |
|  | 1 h |  |  | × |  | × |  |  |  |  |  |
|  | 2 h |  |  | × |  | × |  |  |  |  |  |
|  | 3 h |  | × |  |  |  |  |  |  |  |  |
|  | 4 h | × |  |  |  |  |  |  |  |  |  |
|  | 5 h |  | × |  |  |  |  |  |  |  |  |
|  | 6 h | × |  |  |  |  |  |  |  |  |  |
|  | 7 h |  |  |  |  |  |  |  |  |  |  |
|  | 8 h |  |  |  | × |  | × |  |  |  |  |
|  | 12 h |  |  |  |  |  |  |  |  |  |  |
|  | 20 h |  |  |  | × |  | × |  |  |  |  |
|  | 24 h |  | × |  |  |  |  |  |  |  |  |
|  | 28 h |  |  |  | × |  | × |  |  |  |  |
| Ⅱ | 47 h ~ 48 h | × | × | × | × | × | × | × | × | × | × |
|  | 48 h | Dose Administration | | | | | | | | | |
|  | 48+1 h |  | × |  |  |  |  | × |  | × |  |
|  | 48+2 h | × |  |  |  |  |  |  | × |  | × |
|  | 48+3 h |  |  |  | × |  | × |  |  |  |  |
|  | 48+4 h |  |  |  | × |  | × |  |  |  |  |
|  | 48+6 h |  |  |  | × |  | × |  |  |  |  |
|  | 48+7 h |  | × |  |  |  |  |  |  |  |  |
|  | 48+8 h | × |  |  |  |  |  |  |  |  |  |
|  | 48+12 h |  | × | × |  | × |  |  |  |  |  |
|  | 48+16 h |  |  | × |  | × |  |  |  |  |  |
|  | 48+36 h |  |  | × |  | × |  |  |  |  |  |
|  | 48+48 h | × |  |  |  |  |  |  |  |  |  |
| Ⅲ | 95 h~96 h | × | × |  |  |  |  |  |  |  |  |
|  | 96 h | Dose Administration | | | | | | | | | |
|  | 96 h+0.5h | × |  |  |  |  |  |  |  |  |  |
|  | 96 h+1 h |  | × |  |  |  |  |  |  |  |  |
|  | 96 h+2 h | × |  |  |  |  |  |  |  |  |  |
|  | 96 h+3 h |  | × |  |  |  |  |  |  |  |  |
|  | 96 h+4 h | × |  |  |  |  |  |  |  |  |  |
|  | 96 h+5 h |  | × |  |  |  |  |  |  |  |  |
|  | 96 h+6 h | × |  |  |  |  |  |  |  |  |  |
|  | 96 h+7 h |  | × |  |  |  |  |  |  |  |  |
|  | 96 h+8 h | × |  |  |  |  |  |  |  |  |  |
|  | 96 h+12 h |  | × |  |  |  |  |  |  |  |  |
|  | 96 h+24 h | × |  |  |  |  |  |  |  |  |  |
|  | 96 h+48 h |  | × |  |  |  |  |  |  |  |  |

**Note:** The acceptable time windows for PK/PD sampling were as follows: 0.25, 0.5, 1, and 2 h post-dose (±10 min); 3 and 4 h post-dose (±15 min); 5, 6, 7, and 8 h post-dose (±0.5 h); 12, 16, 20, 24, and 28 h post-dose (±1 h); 36 h post-dose (±1.5 h); and 48 h post-dose (±2 h).

**Supplementary Table S2** PopPK model parameters and bootstrap validation results of nasal lavage fluid

| Parameters | Population typical value | RSE(%) | Shrinkage | Bootstrap test | |
| --- | --- | --- | --- | --- | --- |
|  |  |  |  | median | 95%Cl |
| *k* (1/h) | 0.382 | 7.9 | - | 0.383 | (0.323,0.441) |
| F | 0.00930 | 19.6 | - | 0.00932 | (0.00629,0.0137) |
| IIV *k* | 28.1% | 22.7 | 16% | 28.0% | (16.6%,42.3%) |
| IIV F | 88.9% | 13.7 | 12% | 85.8% | (61.3%,109%) |
| σ_PROP_ | 42.3% | 14.6 | 33% | 41.9% | (26.0%,52.5%) |

**Note:** *k* denotes the elimination rate constant at the nasal lavage compartment level, and F represents the ratio of interferon content in nasal lavage at the instant of administration to the administered interferon dose.

**Supplementary Table S3** PK/PD modeling parameters and bootstrap validation of nasal lavage fluid

| Parameters | Population typical value | RSE(%) | Shrinkage | Bootstrap test | |
| --- | --- | --- | --- | --- | --- |
|  |  |  |  | median | 95%Cl |
| *k*_out_ (1/h) | 0.19 | 38.3 | - | 0.19 | (0.099,0.35) |
| IP10_Base_ (pg/mL) | 140 | 15.0 | - | 135 | (106,185) |
| *E*_max_ | 0.682 | 46.8 | - | 0.679 | (0.217,1.55) |
| EC_50_ (pg/mL) | 112 FIX | - | - | - | - |
| IIV IP10_Base_ | 98.2% | 10.6 | 1% | 96.7% | (76.2%,118%) |
| IIV *E*_max_ | 133.8% | 30.2 | 38% | 133.2% | (85.5%,203%) |
| *σ*_PROP_ | 43.8% | 7.8 | 7% | 43.1% | (36.6%,50.1%) |

**Note:** *k*_out_ is the first-order elimination rate constant of IP10, IP10_Base_ is the baseline value of IP10, *E*_max_ is the maximum effect of interferon in promoting IP10 synthesis, and EC_50_ is the interferon concentration required to achieve half of the maximum effect.

**Supplementary Table S4** Parameter estimates of the E-R relationship model for nasal lavage fluid

| PD indicator | Parameters | estimated value | 95% Cl | P-value |
| --- | --- | --- | --- | --- |
| AUEC/τ | *β*_0_ | 134.42 | (-129.35, 398.19) | 0.3103 |
|  | *β*_1_ | 1.56 | (-0.0988, 3.23) | 0.0646 |
| AUEC_n_/τ | *β*_0_ | 1.04 | (0.673, 1.41) | 8.46e^-07^*** |
|  | *β*_1_ | 0.00276 | (0.000432,0.00508) | 0.0212* |

**Note: ***P<0.05, ***P<0.001.

**Supplementary Fig. S1** **Comparison of IFN and IP10 levels in nasal lavage fluid: initial lavage vs. non-initial lavage.** The bold solid line indicates the population trend, while the gray thin solid lines represent individual trajectories. **(a)** IFN levels. **(b)** IP10 levels.

**
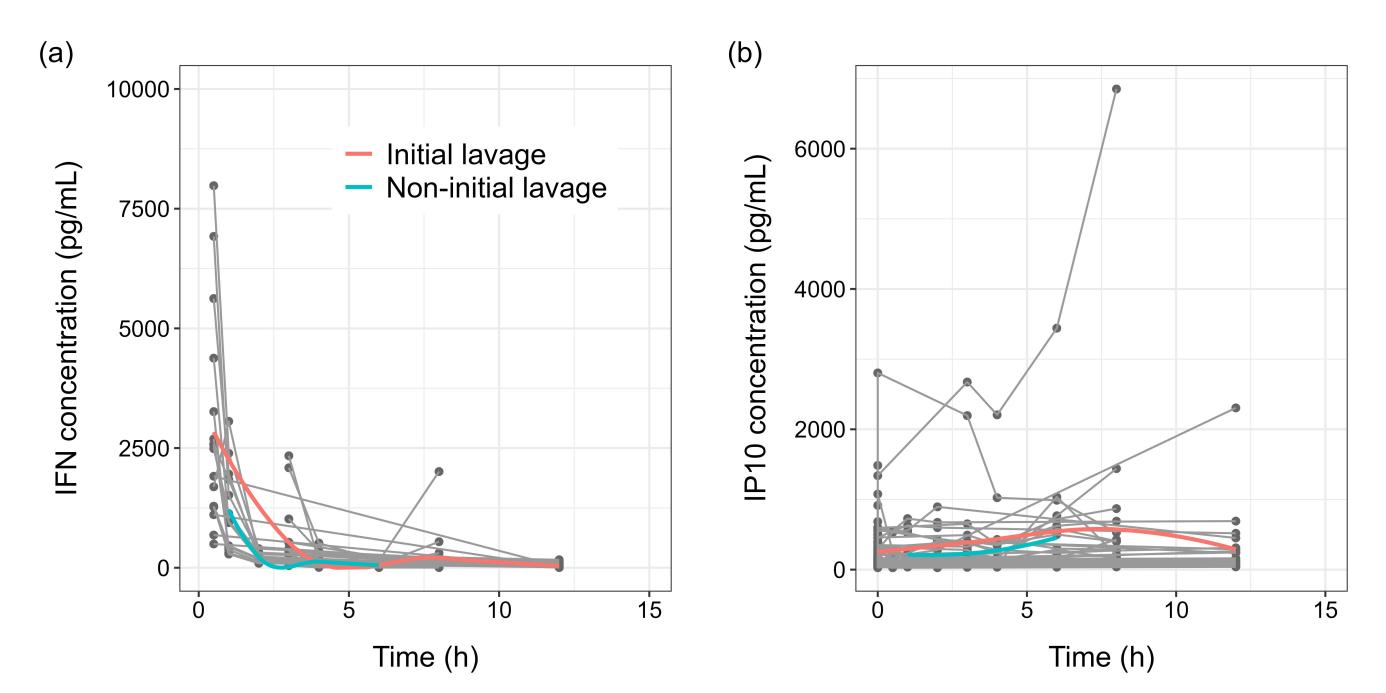
**

**Supplementary Fig. S2 Time course of IFN concentration in logarithmized nasal lavage fluid.**

**
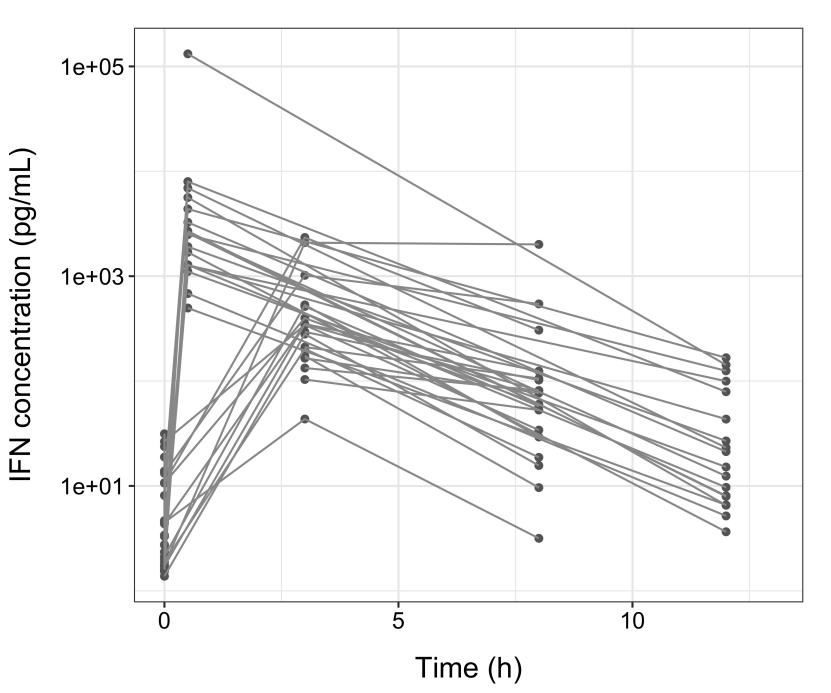
**

**Supplementary Fig. S3** **Blood β2-microglobulin concentration-time curve.** The blue solid lines represent population trend lines. The gray solid lines indicate individual participant profiles.


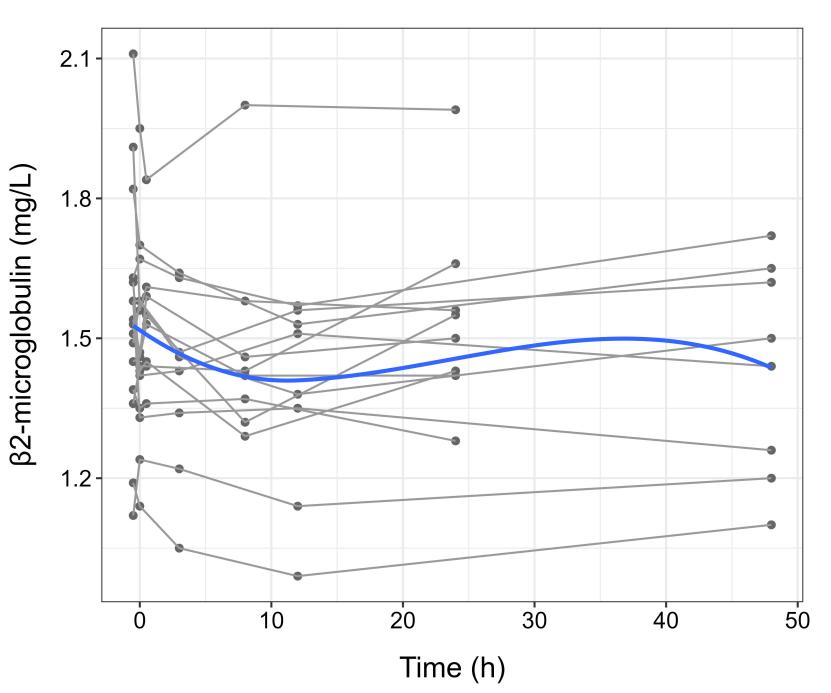


**Supplementary Fig. S4 Concentration-time curve of IP10 in oral gargles.** The blue solid lines represent population trend lines. The gray solid lines indicate individual participant profiles.


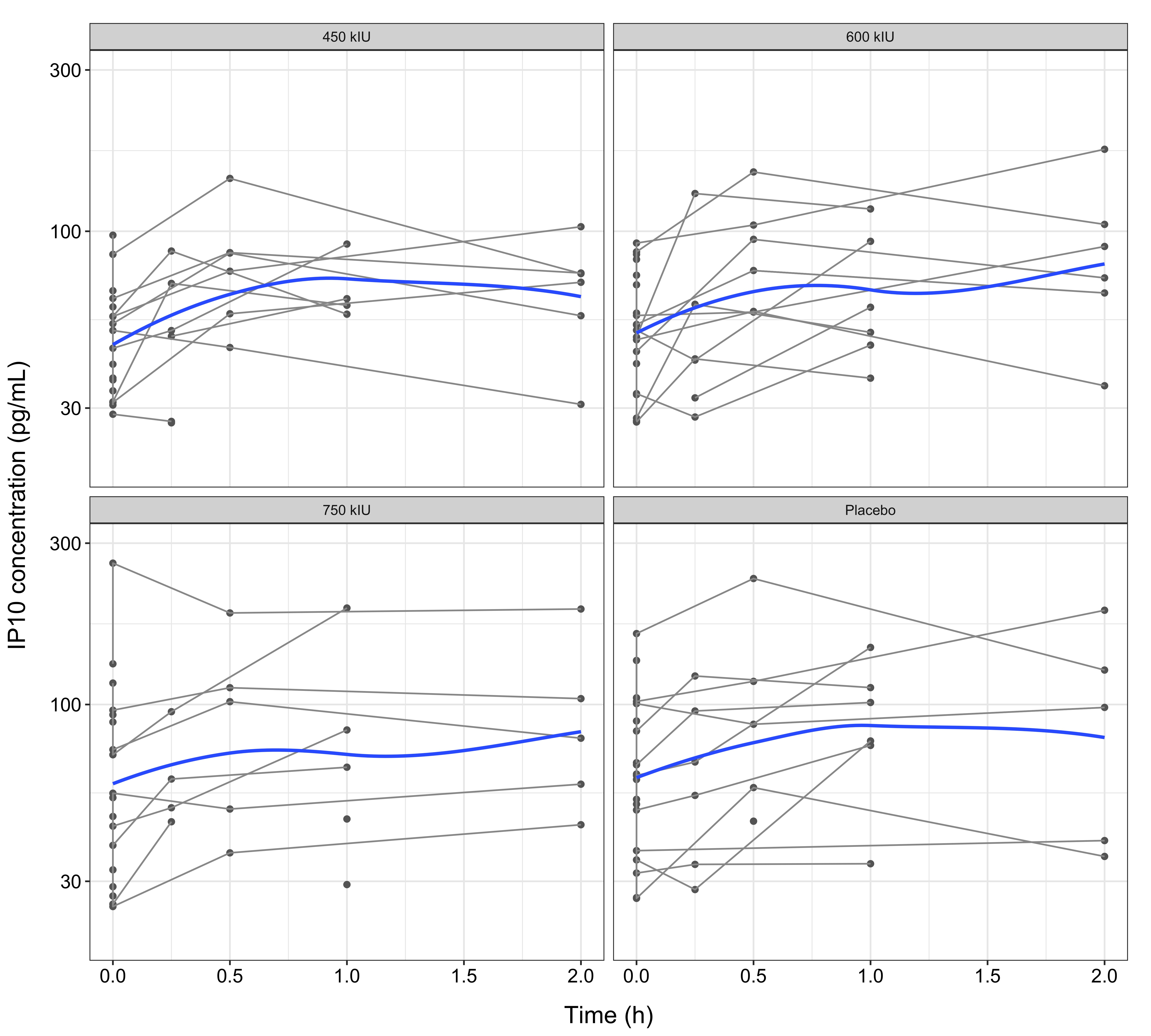


**Supplementary Fig. S5 Block diagram and differential equations. (a)** single-compartment PK model (**b)** IDR model.


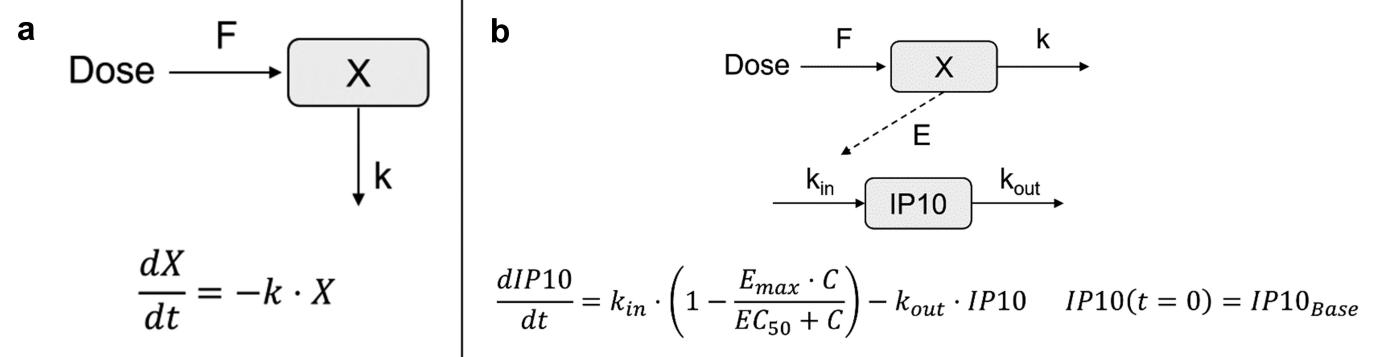


**Supplementary Fig. S6 Model-based simulations of IFN and IP-10 concentration-time profiles in nasal lavage fluid derived from 1,000 virtual participants following a single dose administration**. (**a)** IFN concentration vs. Time; (**b)** IP-10 concentration vs. time. Shaded regions represent 95% prediction intervals, and solid lines indicate median simulated trajectories.


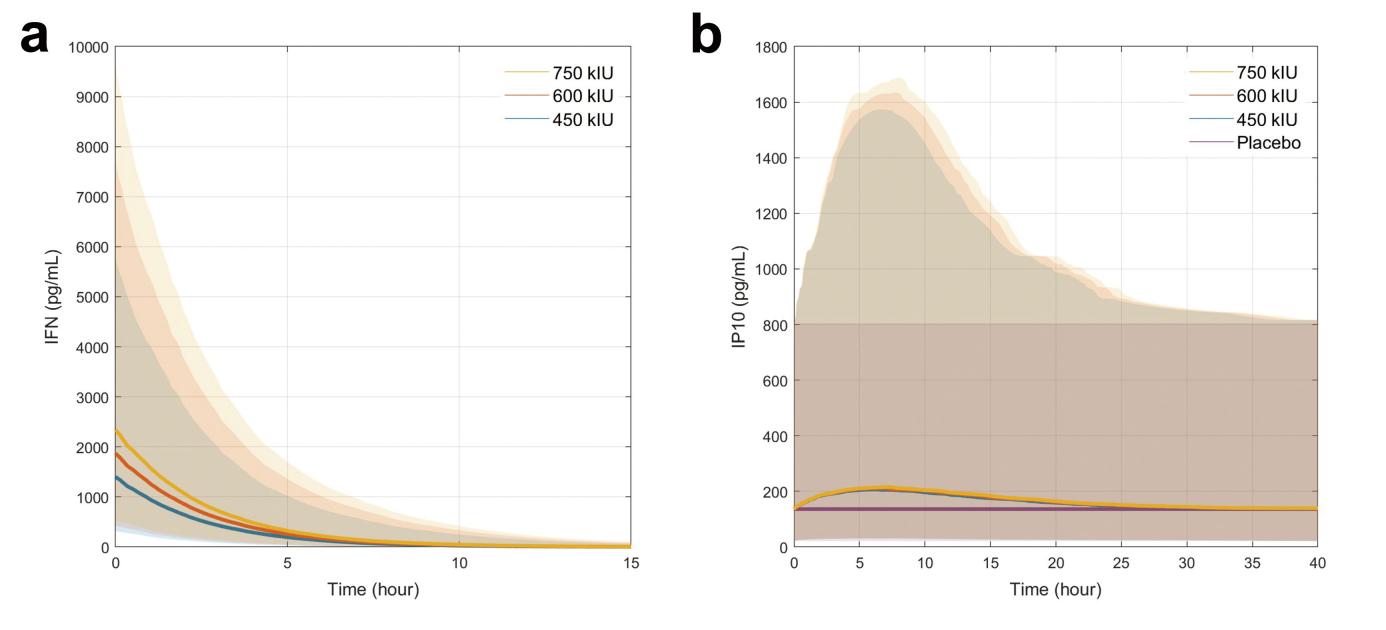

Supplement: Supplemental material — Tables S1 to S4; Fig. S1 to S6. [file aac.00686-25-s0001.docx]
